# Retained Intravesical Needle Following Percutaneous Foley Balloon Puncture: A Rare but Preventable Iatrogenic Complication

**DOI:** 10.7759/cureus.101996

**Published:** 2026-01-21

**Authors:** Pradeep Kumar, Soumya Ghoshal, Navaneeth Pattereth, Ankush Potphode

**Affiliations:** 1 Trauma and Emergency, All India Institute of Medical Sciences, Nagpur, Nagpur, IND; 2 Urology, All India Institute of Medical Sciences, Nagpur, Nagpur, IND

**Keywords:** catheter removal, complication, foley catheter, foreign bodies, non-deflating foley catheter, urinary bladder

## Abstract

Failure of the Foley catheter balloon deflation is often managed using bedside techniques that lack standardisation. We report the case of a 45-year-old man with cervical spinal cord injury who developed fever and reduced urine output following attempted Foley catheter removal at an outside hospital. During percutaneous balloon puncture, the needle fractured and was retained intravesically. Imaging confirmed the presence of a metallic fragment in the bladder. Cystoscopy-guided retrieval was successful. This rare but preventable complication of Foley catheter management emphasises the importance of protocol-based approaches and early specialist involvement when dealing with non-deflating Foley catheters.

## Introduction

Foley catheterisation is one of the most commonly performed procedures in emergency and inpatient settings. Occasionally, removal becomes difficult due to balloon deflation failure, a situation managed at the bedside with various improvised techniques, such as cutting the inflation port, guidewire-assisted deflation, overinflation technique, or instillation of chemical agents [1-3]. When initial measures fail, percutaneous or endoscopic balloon puncture is sometimes attempted as a higher-step intervention, particularly in resource-limited settings. Percutaneous balloon puncture lacks standardisation and carries risks of complications [4].

Intravesical foreign bodies have been reported previously, with most cases described in the literature involving self-insertion or migration [5,6]. In contrast, a focused literature search did not identify well-documented prior reports of intravesical needle retention specifically resulting from percutaneous Foley balloon puncture. Nevertheless, such complications may be under-recognised and under-reported, particularly in resource-limited settings. These events are clinically relevant, as they can lead to urinary retention, infection, and sepsis. We report an unusual case of a retained intravesical metallic needle fragment following percutaneous balloon puncture that presented with febrile episodes. The objective of this case report is to highlight a preventable complication arising from a routine bedside procedure and to emphasise the importance of protocol-based approaches and early specialist involvement in the management of non-deflating Foley catheters.

## Case presentation

A 45-year-old man was referred to our Trauma and Emergency Department with a two-day history of fever and chills and markedly reduced output from his indwelling Foley catheter. He had sustained a cervical spinal cord injury in a road traffic accident six weeks earlier, for which he had undergone surgery and been discharged with an indwelling Foley catheter. While changing the Foley catheter in an outside hospital, attempts to remove it failed because the balloon was non-deflatable. A percutaneous puncture of the balloon with a needle was performed. During the procedure, the needle fractured and was retained, although the balloon deflated and the catheter was removed. A new Foley catheter was inserted. An attempt to retrieve the needle via subcutaneous exploration through a suprapubic incision under local anaesthesia failed to locate the broken fragment. The patient was transferred to our centre four days later.

**Figure 1:**
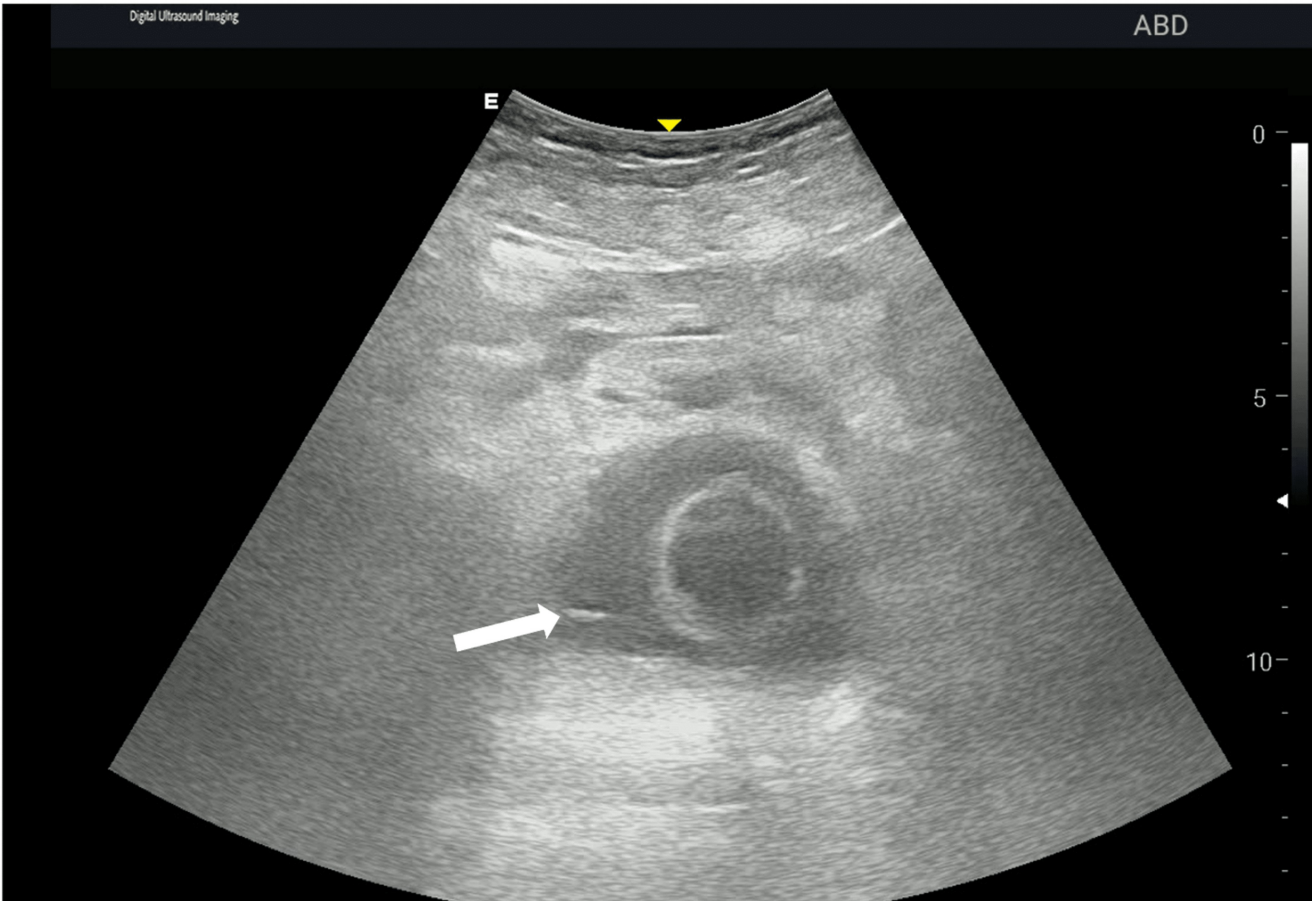
POCUS demonstrating an intravesical foreign body POCUS of the urinary bladder showing a linear echogenic focus within the bladder lumen (white arrow) suggestive of a metallic foreign body. POCUS: point-of-care ultrasound

**Figure 2:**
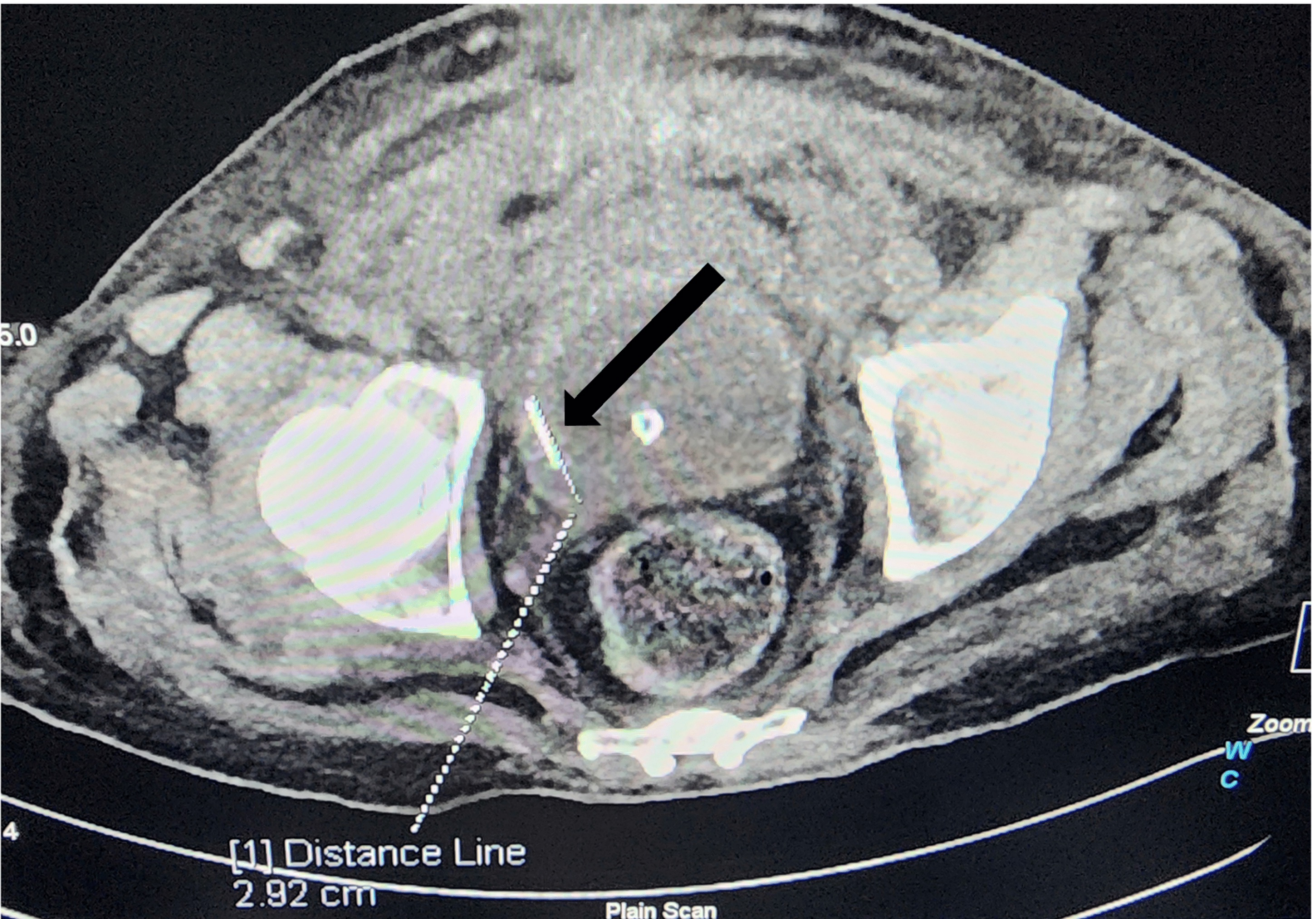
Computed tomography confirmation of an intravesical foreign body Axial non-contrast computed tomography scan of the pelvis demonstrating a linear radio-opaque foreign body within the urinary bladder lumen, adjacent to the right lateral wall (black arrow).

On admission, he was febrile, with a heart rate of 110/min, a blood pressure of 100/65 mmHg, an oxygen saturation of 98% on room air, and a Glasgow Coma Scale (GCS) score of 15 [7]. Neurological examination confirmed quadriparesis with absent sensation below the T4 level and zero power in all four limbs. Inspection revealed bilateral grade II sacral bedsores. A point-of-care ultrasound (POCUS) of the urinary bladder showed a bright, linear echogenic focus, highly suggestive of a metallic needle (Figure 1). A non-contrast computed tomography of the abdomen and pelvis confirmed a radio-opaque needle fragment lying within the urinary bladder lumen, adjacent to the right lateral wall (Figure 2). Based on these findings, a diagnosis of urinary retention with fever and urosepsis secondary to a retained intravesical foreign body was made.

**Figure 3:**
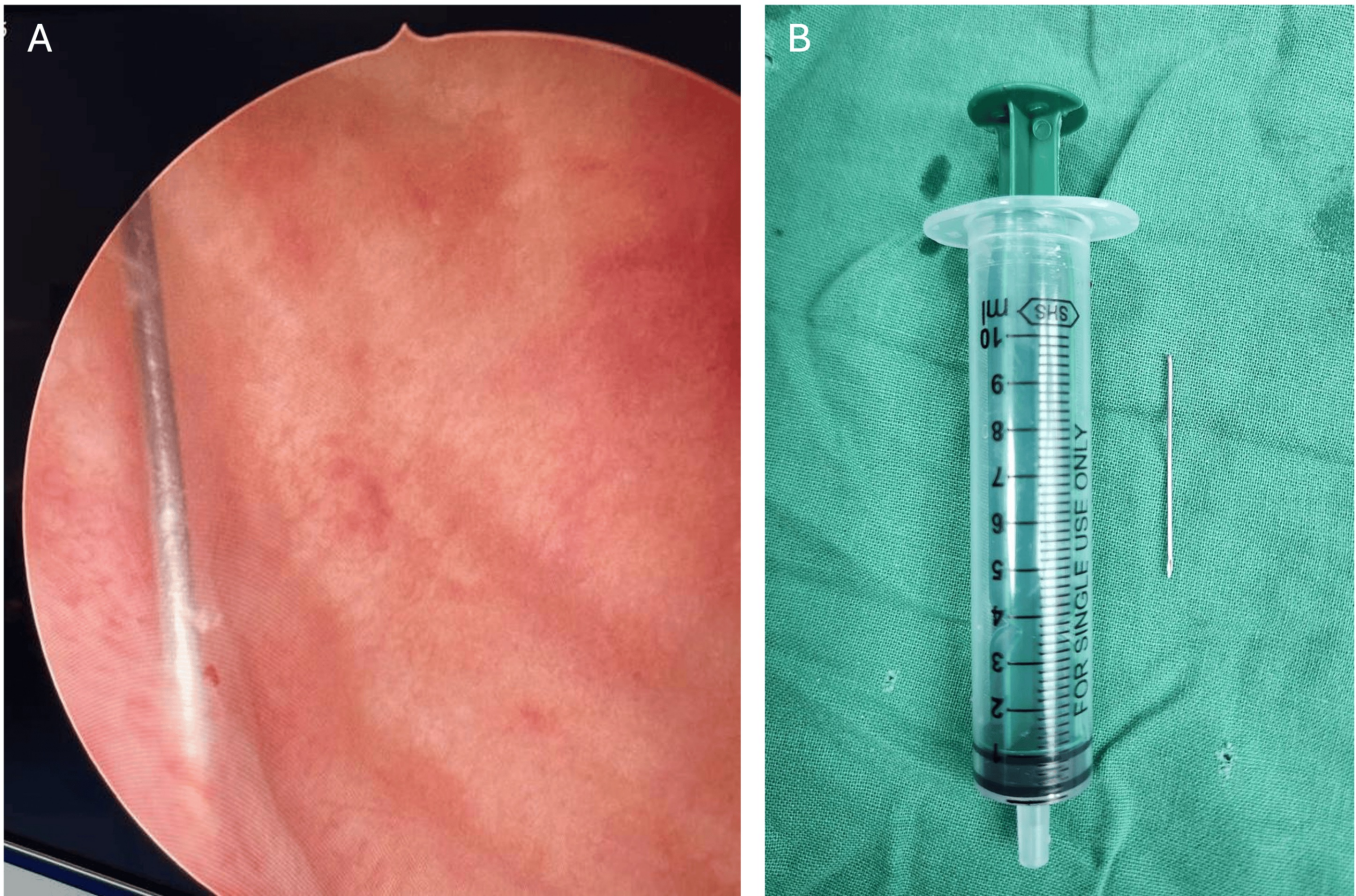
Endoscopic retrieval of the intravesical foreign body (A) Cystoscopic view of the urinary bladder demonstrating a linear metallic foreign body consistent with a needle fragment within the bladder lumen. (B) Retrieved needle fragment following cystoscopic extraction.

Given the confirmed intravesical location of the foreign body and the unsuccessful suprapubic exploration at the primary treating facility, cystoscopy was selected as the safest definitive approach for needle retrieval, avoiding further blind percutaneous attempts. Cystoscopy revealed the broken needle fragment within the bladder (Figure 3A, 3B). The fragment was successfully retrieved using grasping forceps. A Foley catheter was reinserted under vision. The patient became afebrile within 48 hours, with improved urine output and no further catheter-related issues. He was subsequently transferred to the specialised pressure sore care and rehabilitation.

## Discussion

Failure to deflate the Foley catheter balloon is a well-recognised problem. Reported causes include malfunctioning inflation valves, crystallisation within the inflation channel, and external damage to the inflation channel due to clamping or kinking [1]. Several bedside techniques have been described to manage this situation, including cutting the inflation port, passing a guidewire through the inflation channel, or instilling solvents [1-3]. However, many of these methods are based on anecdotal experience rather than standardised protocols. 

When initial methods fail, the balloon puncture technique (transabdominal/suprapubic, transrectal, transperineal, or endoscopic) is used as a higher-step option. Ultrasound-guided percutaneous suprapubic puncture of the Foley balloon is effective, whereas endoscopic (including laser) approaches are reserved when suprapubic puncture fails or is not feasible. Suprapubic puncture carries a risk of complications, particularly when performed in settings with limited access to imaging. 

Although unusual intravesical foreign bodies have been reported previously, this report describes a rarely documented iatrogenic complication of suprapubic Foley balloon puncture [5,6]. To our knowledge, this mechanism of broken needle retention during suprapubic Foley balloon puncture has not been documented in the literature and may be under-reported. However, they are clinically relevant, as they can be prevented through protocol-based escalation and early urological involvement. Intravesical foreign bodies may be self-inserted, trauma-related, or iatrogenic during any per-urethral procedure [5,6]. A retained intravesical foreign body can act as a nidus for infection, leading to urosepsis. Patients with spinal cord injury and neurogenic bladder are particularly vulnerable, as reduced sensation may delay symptom recognition and presentation. 

This case also highlights the diagnostic value of POCUS. In addition to identifying bladder distension, POCUS enabled the early detection of an intravesical foreign body, which was confirmed by computed tomography and allowed definitive planning. Management should aim for safe removal while avoiding additional injury. Endoscopic retrieval remains the preferred management for most intravesical foreign bodies, with open surgery reserved for selected cases [6].

## Conclusions

Non-deflating Foley catheters should be managed with caution. This case provides a valuable lesson about an avoidable complication arising from a common bedside procedure. Blind percutaneous balloon puncture, though commonly attempted, can cause rare but serious complications, including retained intravesical foreign bodies and urosepsis. This case reinforces the importance of protocol-based management of difficult Foley catheter removal, including early imaging and timely urology involvement. Image-guided techniques and cystoscopy-guided removal are preferred over improvised techniques to prevent morbidity.
